# Explaining Racial Inequality in Food Security in Columbus, Ohio: A Blinder–Oaxaca Decomposition Analysis

**DOI:** 10.3390/ijerph17155488

**Published:** 2020-07-29

**Authors:** Keumseok Koh, Michelle L. Kaiser, Glennon Sweeney, Karima Samadi, Ayaz Hyder

**Affiliations:** 1 Department of Geography, University of Hong Kong, Hong Kong, China; 2 College of Social Work, The Ohio State University, Columbus, OH 43210, USA; kaiser.267@osu.edu; 3 Kirwan Institute for the Study of Race and Ethnicity; Department of City and Regional Planning, The Ohio State University, Columbus, OH 43210, USA; sweeney.270@osu.edu; 4 College of Food, Agricultural, and Environmental Sciences, The Ohio State University, Columbus, OH 43210, USA; samadi.2@osu.edu; 5 Division of Environmental Health Sciences, College of Public Health, The Ohio State University, Columbus, OH 43210, USA; hyder.22@osu.edu

**Keywords:** food security, food insecurity, race, inequalities, Blinder–Oaxaca decomposition

## Abstract

Food insecurity is a leading public health challenge in the United States. In Columbus, Ohio, as in many American cities, there exists a great disparity between Black and White households in relation to food insecurity. This study investigates the degree to which this gap can be attributed to differences in food shopping behavior, neighborhood perception, and socioeconomic characteristics. A Blinder–Oaxaca decomposition method is used to analyze a household survey dataset collected in 2014. We find a 34.2 percent point difference in food security between White and Black households. Variables related to food shopping behavior, neighborhood perception, and socioeconomic characteristics explain 13.8 percent, 11.6 percent, and 63.1 percent of the difference, respectively. These independent variables combined can explain 68.2 percent of the food security gap between White and Black households. Most of this is attributable to socioeconomic variables. Sense of friendship in neighborhood, use of private vehicles, and satisfaction of neighborhood food environment also partially contribute to the food security gap.

## 1. Introduction

### 1.1. Overview of Food (In)Security

Food security is a condition under which a person can access adequate, safe, and nutritious food for an active, healthy life without any barriers [1]. To achieve food security, food should be *stably available* over time and people can easily *access* food for one’s nutritional *use*/*utilization* [2,3,4]. The United States Department of Agriculture (USDA) uses four labels to characterize the food security status of households (i.e., all the people who occupy a housing unit regardless of family relations) [5]. Along a continuum, these are *high food security* (i.e., no reported food procurement issues), *marginal food security* (i.e., some anxiety reported on sufficient food procurement, but little or no change in diet or food intake), *low food security* (i.e., reduced diet quality, but little or no reduction in food intake), and *very low food security* (i.e., negative, reduced changes in both diet and food intake) [1].

In 2016, about 12.3 percent of the U.S. population were food-insecure, meaning they had difficulty procuring enough food for all household members at least sometime during the year [6]. Food insecurity may directly result in an unhealthy diet, immediate hunger, and nutritional deficiencies. It is also associated with negative health outcomes in the long term, potentially impairing mobility and ability to work [7,8]. For adults, food insecurity is associated with diet-related chronic diseases including hypertension, diabetes, and obesity, as well as higher mortality [9,10]. Mental health conditions associated with adult food insecurity include depression, anxiety, and social isolation [11,12,13]. For pregnant women, maternal food insecurity is associated with increased risk of certain birth defects and prenatal and postpartum depression [14,15,16]. Food insecurity during childhood and adolescence also contributes to poor health, increased susceptibility to disease, impeded cognitive and physical development, and poor school performance [17,18,19]. 

Importantly, racial and ethnic minority groups in the United States are more vulnerable to household food insecurity [20,21,22,23,24]. In a 2017 USDA report using a nationally representative sample, the percentage of food-insecure Black households (21.8 percent) was 2.5 times higher than that of White households (8.8 percent) [25]. Using 2013 Behavioral Risk Factor Surveillance System collected in 15 U.S. states, Njai et al. [23] found that 68.5 percent (95% confidence interval (CI): 66.3–70.7) of Blacks were food-secure, whereas 81.8 percent (95% CI: 81.2–82.4) of Whites were food-secure. Another study using 2011–2014 National Health and Nutrition Examination Survey found that 17.8 percent (95% CI: 14.3–22.0) of non-Hispanic Blacks were in low food security, while 6.3 percent (95% CI: 5.0–8.0) of non-Hispanic Whites experienced low food security [20].

### 1.2. Risk Factors of Food (In)Security

Multiple factors, independently and interactively, determine food security. First, food security is associated with the socioeconomic characteristics of each person or household. Poverty is closely related to food insecurity: nearly one-third (31.6 percent) of low-income households below 185 percent of the U.S. federal poverty guideline were reported food-insecure in 2018 [6,26,27]. In 2018, the federal poverty level (FPL) was $25,100 for a family of four, so 185% of the FPL was $46,435 [26]. Although the U.S. Supplemental Nutrition Assistance Program (SNAP), formerly known as the Food Stamp Program, provides a monthly allotment of money for food purchases from authorized retailers to eligible participants, SNAP participants are often at a higher risk for food insecurity [28,29].

Lower educational attainment is also associated with a higher chance of food insecurity [6,25]. Single-parent households, living alone, and having children in the household are also related to a higher risk for food insecurity [27,29,30,31,32]. While older adults (≥65 years old) reported being more food-secure in general, additional healthcare costs could put them at risk for being food-insecure [6,33,34].

Second, food shopping behaviors and characteristics such as shopping frequencies, shopping distance, and the access to a private vehicle correlate with food security. Food-insecure households are likely to shop for groceries less frequently [35,36]. When shopping for their food, food-insecure households are more likely to walk and less likely to use their own vehicles [37]. Limited modes of transportation may restrict the total volume of food purchased because it is difficult to carry bulky or heavy items [38]. Food-insecure households often bypass nearby smaller grocery stores and travel farther to large supermarkets that accept SNAP benefits and usually offer lower prices and promotional offers as well as convenient one-stop services like bank tellers, prepared and takeout foods, and pharmacies [39,40,41,42]. However, they may purchase more nonperishable food items than fresh produce, especially when they make a long food shopping trip relying on other persons’ vehicles or public transportation [43,44].

**Figure 1:**
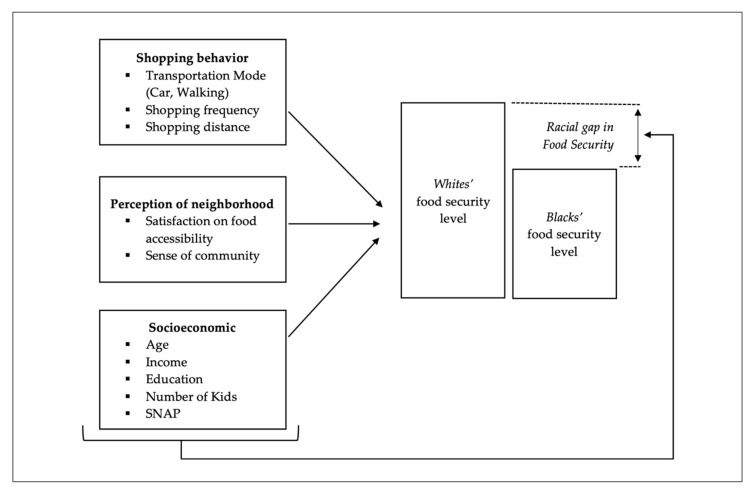
A conceptual model of food security and its determinants. Note. Authors created based on the literature.

Finally, social perception of neighborhood or community is associated with food security. A study conducted in Montreal, Canada, reported that food-insecure households were less satisfied with their food environment in terms of available food options and affordability [45]. The satisfaction of the neighborhood food environment was also related to more fruit and vegetable consumption in predominantly minority neighborhoods in Chicago [46]. Perceived informal social bonds and networks in neighborhoods can reduce the risk of food insecurity by providing additional supports and resources to households living in poor local food environments [47,48]. However, impoverished neighborhoods, especially Black neighborhoods, had relatively weak social bonds and networks in the U.S. [49,50,51]. Figure 1 illustrates a conceptual model of food security based on the literature.

Most quantitative studies using regression models report the roles of individual risk factors on the *odds* of a person or household being food-secure. A typical use of race as an independent variable in such studies is to simply show how much a racial group is more likely to be food-secure compared with a reference group. To address between-groups differentials, however, it would be beneficial for researchers and policymakers to identify how much of the current between-group gap is attributed/not attributed to known risk factors. The Blinder–Oaxaca (BO) decomposition is a well-established analysis method to quantify how much *between-group difference* (e.g., male–female wage gaps, Black–White obesity gaps) can be attributed to known risk factors and unobserved factors, respectively [22]. While originally developed to examine gender and racial group differences in wages, BO analysis has recently gained popularity in population health studies [52].

Using BO approach, this study aims to divide the Black–White food security differential into a part that is “explained” by group differences in socioeconomic characteristics, food shopping behaviors, and neighborhood perception and a remaining part that cannot be accounted for by such differences in the known determinants of food security in Columbus, Ohio [53]. Only one study, to our knowledge, has used this method to investigate the racial food security gap in the U.S. Using a sample obtained in Oklahoma, Nam et al. found that 87 percent of racial inequality in food security can be explained using a set of socioeconomic variables (e.g., income, homeownership, bank account/credit card ownership) and some environmental variables (i.e., metropolitan residency, unemployment rates). Nam et al., however, focused on households with infants [22]. Our study, therefore, is the first to use BO method to investigate racial inequality in food insecurity between a diverse composition of Black–White households from socioeconomic, behavioral, and neighborhood perspectives in a metropolitan U.S. city.

## 2. Material and Methods

### 2.1. Data and Study Area

**Figure 2:**
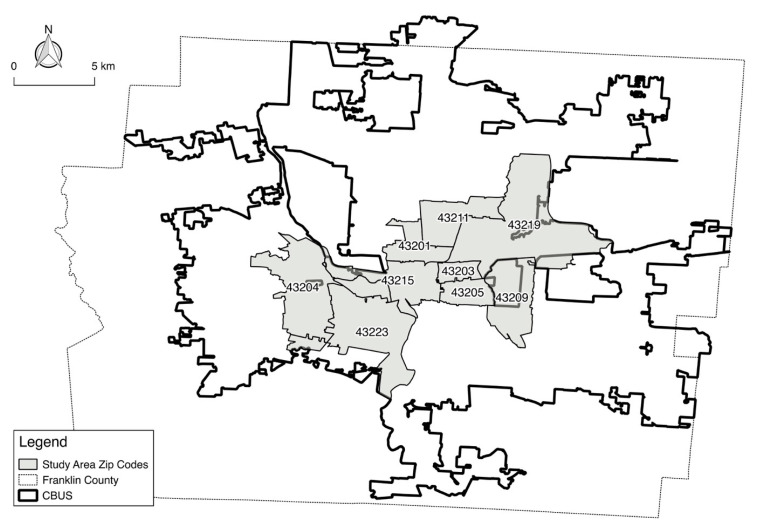
Study area. Note. CBUS: The boundary of the City of Columbus, Ohio.

We used the Ohio State University Food Mapping Team Survey (FMTS) for this analysis. The FMTS was conducted in 2014 via in-person interviews or online surveys in 10 diverse and representative zip code areas in Columbus, Ohio. The study area covers most socioeconomically disadvantaged neighborhoods in Columbus, Ohio. Figure 2 illustrates the study area. The six-part, 88-item questionnaire collected data on food access, food shopping patterns, neighborhood environment, health conditions, food security, and sociodemographic background of respondents and their households [43,54,55]. The total sample size qualified for our study was 586 out of 809 respondents who completed the FMTS. The survey was approved by the Behavioral and Social Sciences Institutional Review Board at The Ohio State University. All respondents were 18 years of age or older and gave informed consent.

### 2.2. Variables

#### 2.2.1. Measure of Food Security

Using the Six-Item Household Food Security Survey Module designed by USDA, the FMTS elicited responses to five questions and statements about respondents’ food procurement experience in the last 12 months [56]: (Q1) “The food that I bought just didn’t last, and I didn’t have money to get more.” Is this statement often, sometimes, or never true for you/your household in the last 12 months? (Q2) “I couldn’t afford to eat balanced meals.” Is this statement often, sometimes, or never true for your situation in the last 12 months? (Q3) In the last 12 months did you or other adults in your household ever cut the size of your meals or skip meals because there wasn’t enough money for food? (Q4) In the last 12 months, did you ever eat less than you felt you should because there wasn’t enough money for food? Finally, (Q5) in the last 12 months, were you ever hungry but didn’t eat because there wasn’t enough money for food? and (Q5-1) if yes, how often did this happen”? A raw score of 1 was given to each response when answered “often” or “sometimes” on questions (Q1) and (Q2), “yes” on (Q3)–(Q5), and “almost every month” and “some months but not every month” on (Q5-1). Households with a row score of 2 or greater (maximum of 6) were considered as food-insecure.

#### 2.2.2. Independent Variables

We used three types of independent variables from FMTS for analysis: First, a set of food shopping behaviors of household (i.e., modes of transportation, monthly shopping frequencies, and mean shopping distance to a food store) were included in the model. Second, the variables describing the social perception of neighborhood reported by the FMTS respondents were included in the model. Nasar and Julian [57]’s questionnaire surveying sense of community was used to obtain scaled responses to four survey questions and statements as follows: (1) “How satisfied are you with the ease of which you can access the food you want to eat in your neighborhood?” (2) “My friends in the neighborhood are part of my everyday activities”, (3) “People here know they can get help from others in the neighborhood if they are in trouble”, and (4) “I have NO friend in the neighborhood on whom I could depend.” And finally, socioeconomic characteristics related to households’ food security (i.e., educational attainment, household income, the number of children in the household, and participation in the SNAP) were included in the model.

### 2.3. Statistical Analysis

We conducted a BO decomposition analysis to investigate the contribution of each independent variable to racial differences in food security [58]. The mean group-specific food security rates for Whites ($\bar{{\mathrm{FS}}^{W}}$) and Blacks ($\bar{{\mathrm{FS}}^{B}}$) can be denoted as follows:

(1) $$\bar{{\mathrm{FS}}^{W}}={E_{ß}}^{W}({Y_{i}}^{W}|{X_{i}}^{W})$$

(2) $$\bar{{\mathrm{FS}}^{B}}={E_{ß}}^{B}\;({Y_{i}}^{B}|{X_{i}}^{B})$$

where Y_i_ is a regression models to estimate food security, X_i_ is a vector of i covariates, and E_ß_(Y_i_|X_i_) is the conditional expectation of Yi estimated at the parameter vector ß. The food security gap between Whites and Blacks, Δ, can be expressed as follows:

(3) $$\begin{array}{c} \Delta =\bar{{\mathrm{FS}}^{W}}-\bar{{\mathrm{FS}}^{B}}\\ ={E_{ß}}^{W}({Y_{i}}^{W}|{X_{i}}^{W})-{E_{ß}}^{B}({Y_{i}}^{B}|{X_{i}}^{B})\\ =[{E_{ß}}^{W}({Y_{i}}^{W}|{X_{i}}^{W})-{E_{ß}}^{W}({Y_{i}}^{B}|{X_{i}}^{B})]+[{E_{ß}}^{W}({Y_{i}}^{B}|{X_{i}}^{B})-{E_{ß}}^{B}({Y_{i}}^{B}|{X_{i}}^{B})] \end{array}$$

where E_ß_^W^(Y_i_^B^|X_i_^B^) refers to a hypothetical term with the mean X values of the Blacks and the coefficient ßs of the Whites. The term assumes a hypothetical condition that Blacks have the same characteristics in all covariates (i.e., the effect of education is the same in both racial groups). In Equation (3), the first term on the right side is considered the “explained” portion of the gap, because the differential of the two groups is due to group differences in levels of covariates. In contrast, the second term is the “unexplained” portion due to differences in coefficients between the two groups [52,53,59]. In other words, the mean values of covariates and their regression coefficients for each group are involved in the decomposition [54].

For analysis, we constructed a set of four logit models. In all four, the status of being food-secure (high or marginal food security = 1, low or very low food security = 0) was the dependent variable. Model 1, 2, and 3 analyzed shopping behavior, neighborhood perception, and socioeconomic variables, respectively; Model 4 incorporated all three. Analyses were conducted using “*Oaxaca*” command in *Stata SE 14.1* [54,60]. The categorical variables in the model were transformed as a set of dummy variables for the use of “*Oaxaca*” command since the decomposition results could change with the choice of reference categories [54].

## 3. Results

Table 1 provides the descriptive statistics of the two racial groups. Compared with Black households, White households used their own cars more often and walk less to buy food, shopped 1.4 times more frequently, and travelled 0.6 miles less to acquire food in one month. Regarding neighborhood perception, White households were more satisfied with food accessibility in their neighborhood, and had more perceived connections with friends and neighbors in their communities. In terms of socioeconomic characteristics, White respondents were younger, possessed higher educational and household income levels, had fewer children in their homes, and were less likely to participate in SNAP.

Table 2 shows the explained and unexplained portions of the food security gap between Black and White households. There was a 34.2 percent point (95% CI: 25.4–43.1) difference in food security between White (75.6%, 95% CI: 71.9–79.9) and Black (41.6%, 95% CI: 33.7–49.5) households.

Model 1 shows racial inequality in food security explained by shopping behavior and characteristics. Only 13.8 percent (95% CI: 4.2–23.3) of the gap was explained by the shopping behavior variables—monthly shopping frequency, mode of travel (use of own vehicle, walking), and mean distance to shopping destination in combination; the remaining 86.2 percent (95% CI: 59.5–113.0) remained unexplainable by differences in shopping behavior. Shopping by own vehicle was the only variable independently explaining the racial gap in food security with statistical significance.

Model 2 presents how much of the racial gap in food security can be explained with perception of neighborhood. Only 11.6 percent (95% CI: 3.7–19.5) of the gap was explained with the satisfaction of food environment in neighborhood, sense of connection with friends in the neighborhood, of having help from neighbors, and of having no friends in the neighborhood. Notably, sense of having no friends in the neighborhood was the most statistically significant in explaining the racial food security gap.

Model 3 summarizes the extent to which socioeconomic variables explain racial inequality in food security. These variables explained 63.1 percent (95% CI: 46.2–80.0) of the gap. Educational attainment level, household income, the number of children in the household, and participation in SNAP were all statistically significant in explaining racial inequality in food security.

Model 4 reports the results when using all three types of variables together. While 68.2 percent (95% CI: 50.4–85.9) of the racial food security gap was explained by these variables, 31.8 percent (95% CI: 3.8–60.0) remained unexplained. Satisfaction with neighborhood food environment, sense of no friends in the neighborhood, education, household income, number of children, and participation in SNAP were statistically significant in this model.

## 4. Discussion

As summarized in Table 1, Black households in the study area have more disadvantaged socioeconomic and environmental conditions. These respondents are older, less educated, and have a lower income compared with White respondents. While traditionally the study area has been predominantly Black, neighborhood change through gentrification and the growth of a local state university has drawn in young White residents. Differences in socioeconomic conditions may further explain differences in shopping behavior. Higher levels of education and income in White households may contribute to easier access to private vehicles in which to go shopping and higher shopping frequency. Easier access to personal vehicles could also mean that White households are able to choose to shop at supermarkets or grocery stores with consistently lower prices, discounts, and deals and, therefore, are less likely to report being food-insecure compared with Black households [61]. There are also differences in food environment and perceptions of food environment. Black households travel further to purchase food, implying there may be fewer food stores with healthy food in their neighborhood. Therefore, it is unsurprising that Black households are less satisfied with their food environment in the study area. The additional distance travelled by Black households to acquire food would also increase the total cost of food shopping (i.e., additional gas, public transportation fare, time spent traveling), which is yet another example of how the food environment may be associated with the racial gap in food insecurity rates [62].

**Figure 3:**
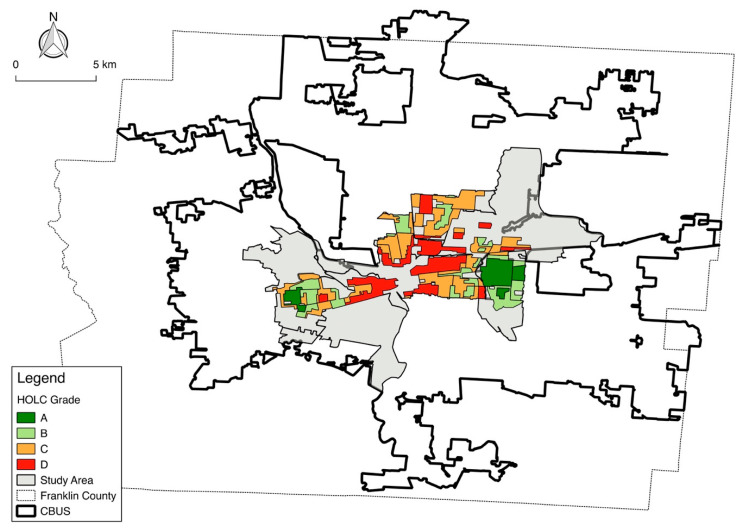
A Home-Owners’ Loan Corporation (HOLC) redlining map in Columbus, Ohio, in 1936. Note. CBUS: the boundary of the City of Columbus, Ohio. Source: Redlining Maps—Ohio Cities: Ohio State University Libraries: http://guides.osu.edu/maps-geospatial-data/maps/redlining/. Authors re-created.

Among the three categories of variables, the majority of the food security gap between White and Black households is attributable to differences in individual socioeconomic variables. We find a distinct inequality in household income and educational attainment between Black and White households, and this is the primary factor driving the food security gap between the two racial groups. When examining such racial disparities, it is critical to consider the historical impact of policy in driving the disparities. Columbus ranks 78 out of the 318 most racially segregated cities in the U.S. [63]. The racial segregation of American cities is a cumulative result of policies enacted throughout the twentieth century. For example, most historically Black communities received “D” ratings from the Home-Owners Loan Corporation (HOLC), redlining these communities (Figure 3) [64]. Redlining meant that these communities were ineligible to receive federally insured mortgages for the purchase or refinancing of property, resulting in disinvestment and “blight.” Black people in Columbus and across the U.S. were barred from purchasing property in communities with higher HOLC grades due to the prolific use of racially restrictive covenants, conventions written into home deeds which prohibited the sale of properties to Black people and other ethnic and religious minority groups [65]. In Ohio, schools are funded by local property taxes, resulting in unequal funding allocations to school districts in wealthier versus less wealthy neighborhoods. Due to policies like redlining, many communities of color have experienced decades of disinvestment, resulting in lower property values and less money to fund public education. Policies that fund public schools equitably by either using revenue from sources other than property taxes or aggregating and redistributing property taxes at the state level could address some of the educational disparities found in the study area. The impact of redlining and racially restrictive covenants on wealth generation in redlined communities is still visible today through the racial gap in food security.

The long-term impacts of practices like redlining which led to disinvestment in many minority urban communities are found not only in blighted built environments, but also in the social fabric of communities. We find that social networks in neighborhoods are a strong predictor of the Black–White gap in food security. Satisfaction with neighborhood food environment also partially explains the racial gap in food security when considering all variables. These findings imply that improving neighborhood conditions may be an effective strategy to improve food security by building social networks and self-supports [46]. For example, implementing community-based gardening projects could be an effective neighborhood-based intervention to provide additional access to fresh vegetables and build healthier family and social relationships [66]. Also notable is the “corner store intervention”, a response to major supermarket chains’ tendency to relocate existing stores from impoverished inner-city neighborhoods to affluent suburbs or unwillingness to open new stores in poor areas [67]. The goal of the intervention is to provide additional fresh fruit and vegetables and other healthy food to local stores and upscale the store arrangement and display to promote increased consumption of healthy food [68]. The findings of our study suggest that the corner store intervention would be more effective if it also aimed to enhance the sense of community among residents by increasing daily contact and friendships between neighbors through shopping in these spaces.

When considering only shopping behavior variables, it is notable that using one’s own vehicle to go shopping is attributable to the racial gap in food security. This finding reiterates the lower socioeconomic status of Black households and also supports the food security literature stressing the importance of transportation mode [35,36]. However, this effect becomes statistically not significant when analyzed with all the variables in this study (Model 4), implying that the differences in transportation mode alone may not lead to a large racial gap in food security when considering a variety of the other predictors of food security.

Receiving SNAP is an important factor in explaining the racial gap in food security because more Black households (46.6%) than White (13.2%) participate in SNAP in our study. This implies that SNAP may be insufficient to make a household food-secure. Leung et al. also found that SNAP participation does not necessarily lead to food security, compared with non-participants [69]. While SNAP substantially helps food-insecure households, there is criticism regarding its benefits for individual health and well-being. For example, while the average meal cost in the U.S. is $2.36, the SNAP benefit per meal is $1.86 [70,71]. For households with children, it is calculated even lower, at $1.40 [72]. Further research, therefore, is needed to focus on the dynamics of disposable income and food purchase for food security by incorporating other sources of income, social welfare programs, and spending for other purposes such as medical treatment, housing, and education as well as the appropriate level of the SNAP benefit and the budget [43].

While our study illuminates the drivers of the racial food gap in Columbus, Ohio, it has some limitations. First, although the study uses comprehensive information on household food security, this information was collected from a cross-sectional survey. Regularly performed surveys in a wider range of geographical areas would help to inform a more insightful analysis. Second, the data used in the study was collected via self-reporting. Future research is needed to design more comprehensive methods to quantitatively measure household food security and neighborhood characteristics for analysis. Leroy et al. [73] suggest that more robust food security indicators based on accessibility, details of food consumption, and more information about nutritional value may reveal more relevant findings for food security inequalities. Third, while this study identifies the extent to which socioeconomic variables, neighborhood perception, and shopping behavior contribute to the racial gap in food security, it does not aim to find new contributing variables. More research is needed to focus on finding new variables that could potentially explain the substantial portion of the racial food security gap that remains unexplained. For example, future studies can utilize more socioeconomic (e.g., amount of SNAP benefit, number of employed people per household, amount of healthcare spending) and/or environmental (e.g., availability of food items or community food assistance programs, walkability) determinants of households. It is also possible that structural racism could help explain the unexplained portion of the racial food security gap. Qualitative research exploring this notion could shed light on these disparities by providing more insight into the extent of barriers to social capital, such as discrimination and racism in the food environment.

## 5. Conclusions

This study uses the BO decomposition method to partition the gap in food security between Black and White households in Columbus, Ohio, into explained and unexplained portions with independent variables. We analyze a survey on food security status, shopping behavior, neighborhood, and socioeconomic characteristics, finding that the majority of the racial gap in food security is attributable to socioeconomic characteristics of households. Poor neighborhood social network is an important factor in explaining the gap. We also find that use of a private vehicle for food shopping and satisfaction with neighborhood food environment partially contribute to the racial food gap. However, a substantial portion of the racial food security gap remains unexplained by the variables used in this study. To reduce racial disparities in food security, future policy interventions should address differences in variables, especially levels of educational attainment and household income. Specifically, community leaders can seek opportunities to distribute free and affordable produce through the local foodbanks and expand SNAP and WIC (Special Supplemental Nutrition Program for Women, Infants, and Children) benefits to lessen the financial burden on low-income households. Future studies are also needed to explore additional variables that potentially contribute to the racial gap in food security.

## Figures and Tables

**Table 1:** Descriptive statistics.

Variables	Blacks	Whites
***Shopping Behavior***		
**Shop by own car (%)**		
Never (ref.)	34.46	14.12
Sometimes	6.08	9.79
Most	59.46	76.08
**Shop by walking (%)**		
Never (ref.)	52.70	41.23
Sometimes	34.46	48.52
Most	12.84	10.25
**Mean times shopped for food per month ^#^**	5.36	6.70
**Mean shopping distance (miles) ^#^**	4.50	3.87
***Perception of Neighborhood***		
**Satisfied with neighborhood food accessibility (%)**		
Never (ref.)	23.47	16.03
Somewhat	37.76	42.57
Very	38.78	41.40
**Connections with friends (%)**		
Strongly disagree (ref.)	29.73	12.53
Disagree	34.46	35.08
Not sure	6.08	7.97
Agree	21.62	31.89
Strongly agree	8.11	12.53
**Neighbors’ recognition on help availability (%)**		
Strongly disagree (ref.)	18.24	7.74
Disagree	14.19	15.72
Not sure	22.30	31.66
Agree	33.11	33.03
Strongly agree	12.16	11.85
**No friend in neighborhood (%)**		
Strongly disagree (ref.)	20.27	36.67
Disagree	35.14	37.13
Not sure	8.11	6.61
Agree	18.92	12.07
Strongly agree	17.57	7.52
***Socioeconomic***		
**Age group (%)**		
18–24 (ref.)	12.84	15.95
25–34	11.49	33.26
35–44	18.24	16.86
45–54	20.95	16.86
55–64	24.32	11.39
65+	12.16	5.69
**Education (%)**		
High school (ref.)	38.51	9.34
Some college	37.16	24.37
College	11.49	33.26
College+	12.84	33.03
**Household income (%)**		
25K–(ref.)	65.54	31.66
25–34K	8.78	9.34
35–50K	7.43	13.21
50–74K	10.81	15.03
75–100K	4.05	9.34
100K+	3.38	21.41
**Number of kids (%)**		
0 (ref.)	63.51	80.18
1	15.54	10.71
2	7.43	4.56
3	13.51	4.56
SNAP (%) *		
Yes (ref.)	46.62	13.21
No	53.38	86.79
**Total sample size**	148	438

Note. SNAP: Supplemental Nutrition Assistance Program (SNAP). ref.: reference categories; ^#^ continuous variables; * dummy variable; otherwise, categorical variables.

**Table 2:** Explained and unexplained portions of food security gap between Blacks and Whites.

	Race/Ethnicity	Mean Food Security/Gap				95% CI				*p* > z			
	A. Non-Hispanic White (*n* = 438)	75.85%				71.85, 79.86				<0.000			
	B. Non-Hispanic Black (*n* = 148)	41.61%				33.69, 49.53				<0.000			
	Black–White Gap (A–B)	34.24%				25.37, 43.12				<0.000			
		**Model 1**			**Model 2**			**Model 3**			**Model 4**		
	**Explanatory Variables**	**% Explained**	**95% CI**	***p* > z**	**% Explained**	**95% CI**	***p* > z**	**% Explained**	**95% CI**	***p* > z**	**% Explained**	**95% CI**	***p* > z**
Shopping	Shop by own car	12.76%	4.61, 20.91	0.002							0.91%	−5.02, 6.86	0.764
	Shop by walking	−0.85%	−2.86, 1.17	0.409							−1.34%	−3.71, 1.05	0.273
	Monthly shopping frequency	1.64%	−1.58, 4.88	0.317							0.00%	−2.83, 2.83	0.999
	Mean shopping distance	0.20%	0.85, 1.26	0.697							0.12%	−0.53, 0.73	0.745
Neighborhood	Satisfied with neighborhood food accessibility				2.63%	−0.58, 5.87	0.110				2.66%	−0.47, 5.78	0.096
	Connections with friends				−1.99%	−7.27, 3.27	0.458				−0.67%	−5.61, 4.29	0.793
	Neighbors’ recognition on help availability				0.88%	−1.40, 3.15	0.455				0.96%	−1.29, 3.21	0.397
	No friend in neighborhood				10.08%	2.60, 17.58	0.008				5.58%	−0.41, 11.54	0.068
Socioeconomic	Age							−2.45%	−7.68, 2.75	0.355	−1.17%	−6.54, 4.21	0.669
	Education							17.84%	6.37, 29.32	0.002	18.08%	6.92, 29.26	0.002
	Household income							23.60%	14.16, 33.03	<0.000	20.97%	11.74, 30.23	<0.000
	Number of kids							5.05%	0.09, 10.05	0.047	5.20%	0.26, 10.11	0.039
	SNAP							19.01%	7.24, 30.81	0.002	16.85%	5.14, 28.53	0.005
	Total difference explained	13.76%	4.23, 23.31	<0.000	11.59%	3.68, 19.51	0.004	63.05%	46.17, 79.96	<0.000	68.17%	50.44, 85.89	<0.000
	Total difference unexplained	86.24%	59.49, 113.00	<0.000	88.41%	62.00, 114.84	<0.000	36.95%	9.14, 64.72	0.009	31.83%	3.80, 59.87	0.026
